# Medication adherence to secondary prevention after ischemic cerebrovascular disease: a real-world outcomes analysis

**DOI:** 10.3389/fneur.2026.1764948

**Published:** 2026-03-06

**Authors:** Sabrina M. Stollberg, Andri Signorell, Andreas R. Luft, Carola A. Huber

**Affiliations:** 1 Department of Health Sciences, Helsana Group, Zurich, Switzerland; 2 Department of Neurology, University Hospital Zurich, University of Zurich, Zurich, Switzerland; 3 Cereneo Center for Neurology and Rehabilitation, Vitznau, Switzerland; 4 Institute of Primary Care, University Hospital Zurich, University of Zurich, Zurich, Switzerland

**Keywords:** cardiovascular outcome, ischemic cerebrovascular disease, medication possession ratio, secondary prevention, stroke recurrence

## Abstract

**Introduction:**

Ischemic cerebrovascular disease (ICD) poses a major global burden. Non-adherence to medical secondary prevention leads to recurrent events and disability. Real-world data on adherence to preventive medications is scarce. The objective here is to determine adherence to secondary prevention of ICD and its effect on clinical outcomes.

**Methods:**

This retrospective observational study was based on claims data from a large Swiss health insurance. We studied patients aged 18 years or older, who were hospitalized for ICD between 2017 and 2021. Patients’ one-year medication adherence was determined by the medication possession ratio (MPR, high adherence defined as MRP ≥ 0.80). Outcome variables were all-cause death, recurrent stroke, admission to nursing home, and major adverse cardiovascular events.

**Results:**

A total of 9,911 patients with ischemic stroke or TIA were included in the analysis. Lipid-lowering drugs (LLD) had the largest proportion of high adherence users (63.2%), followed by antihypertensives (55.4%) and antiplatelets (50.0%). Female patients were 37% less likely to adhere to LLD therapy than men, highlighting a significant gender gap. Users with high adherence to LLD had a significantly reduced risk for all-cause death (HR 0.86, 95% CI 0.79, 0.94); Direct oral anticoagulants (HR 0.88, 95% CI 0.78, 1.00) and antihypertensives (HR 0.93, 95% CI 0.85, 1.01) showed a trend towards a protective effect.

**Discussion:**

A remarkable proportion of patients were non-users or had low adherence to medical secondary prevention. Since insufficient secondary prevention may lead to an increased all-cause death rate, efforts are needed to raise awareness among healthcare professionals and improve patient compliance.

## Background

Ischemic cerebrovascular disease (ICD) is a major global burden of disease. The worldwide lifetime risk of ischemic stroke after the age of 25 years was estimated to be as high as 18.3% (1). In a recent meta-analysis, the pooled risk of stroke recurrence at two years was 16% (2). Studies describe the associated risk of increased mortality, stroke recurrence and adverse outcome upon cessation of secondary prevention (3, 4). There is evidence that 45–80% of recurrent transient ischemic attacks and strokes could be prevented (5, 6). Therefore, current international guidelines agree on the importance and extent of medical secondary prophylaxis with antithrombotic, lipid lowering and antihypertensive agents to prevent further disease burden (7–9). However, a meta-analysis in 2016 suggests that adherence to secondary prevention is not optimal with a non-adherence rate of around 31% (10). In an Australian retrospective cohort study of 9,817 adults with first-ever stroke or TIA up to one-third of the patients discontinued secondary preventative medication over the subsequent year post discharge (11). To the best of our knowledge the rate of adherence to medical secondary stroke prevention has not been evaluated in Switzerland to date. Moreover, there is limited international evidence based on real world data when it comes to current and comprehensive overviews with both a high-standing methodology and analyses of (1) all drug classes recommended for secondary stroke prevention and (2) different clinical outcomes, e.g., all-cause death, restroke, major adverse cardiovascular events (MACE) or admission to nursing homes. Therefore, we aimed to determine the medical adherence for secondary prevention after ICD and to access its influence on clinical outcomes in a real-world setting based on healthcare claims data.

## Materials and methods

### Study design, data sources, and study population

This was a retrospective cohort study using healthcare claims data from the Helsana Group (Helsana). Helsana is one of the largest health insurance companies in Switzerland covering about 1.4 million mandatory insured persons, which corresponds to around 15% of the Swiss population.

**Figure 1:**
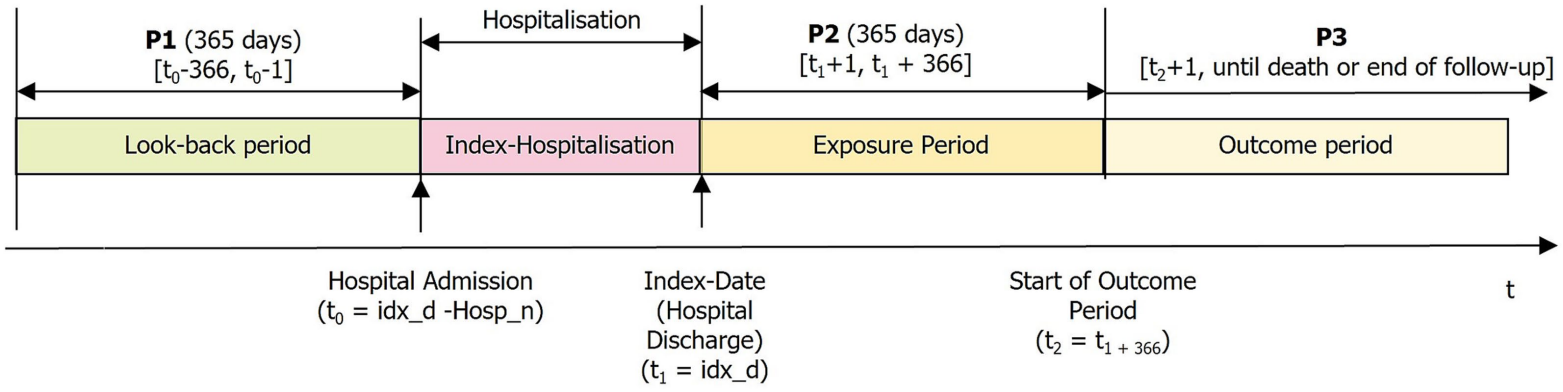
Study design: timeline.

We included men and women aged 18 years or older with mandatory health insurance at Helsana who were hospitalized for ICD between January 1, 2017, and December 31, 2021. Diagnosis of ICD was classified according to the International Classification of Diseases, 10th revision, German modification (ICD-10-GM-2023) (12). Patients with the codes I63 (ischemic stroke), I64 (stroke, not specified as haemorrhage or infarction) and G45 [transitory ischemic attack (TIA)] were eligible. Patients who were not insured with Helsana or had a stroke in the look-back period were excluded. Patients who died during the index hospitalization or died or dropped out of the insurance within the first year after hospital discharge were also excluded, as were patients who had a recurrent stroke or MACE during the exposure period (Figure 1).

### Exposure assessment

Medical adherence was operationally defined by the medication possession ratio (MPR). The MPR was calculated by dividing the number of days of medication prescribed based on Defined Daily Doses (DDD) (numerator) by 365 days (denominator) (13).

$$\mathrm{MPR}={\text{Days}}^{’}\;\text{supply}\;(\mathrm{DDD})/365\;\text{days}$$

DDD were adopted from World Health Organization Collaborating Centre (WHOCC) for Drug Statistics Methodology (14). *Days’ supply* was calculated by dividing the total prescribed quantity of the substance per patient and per 365 days in grams by the corresponding DDD in grams.

**Figure 2:**
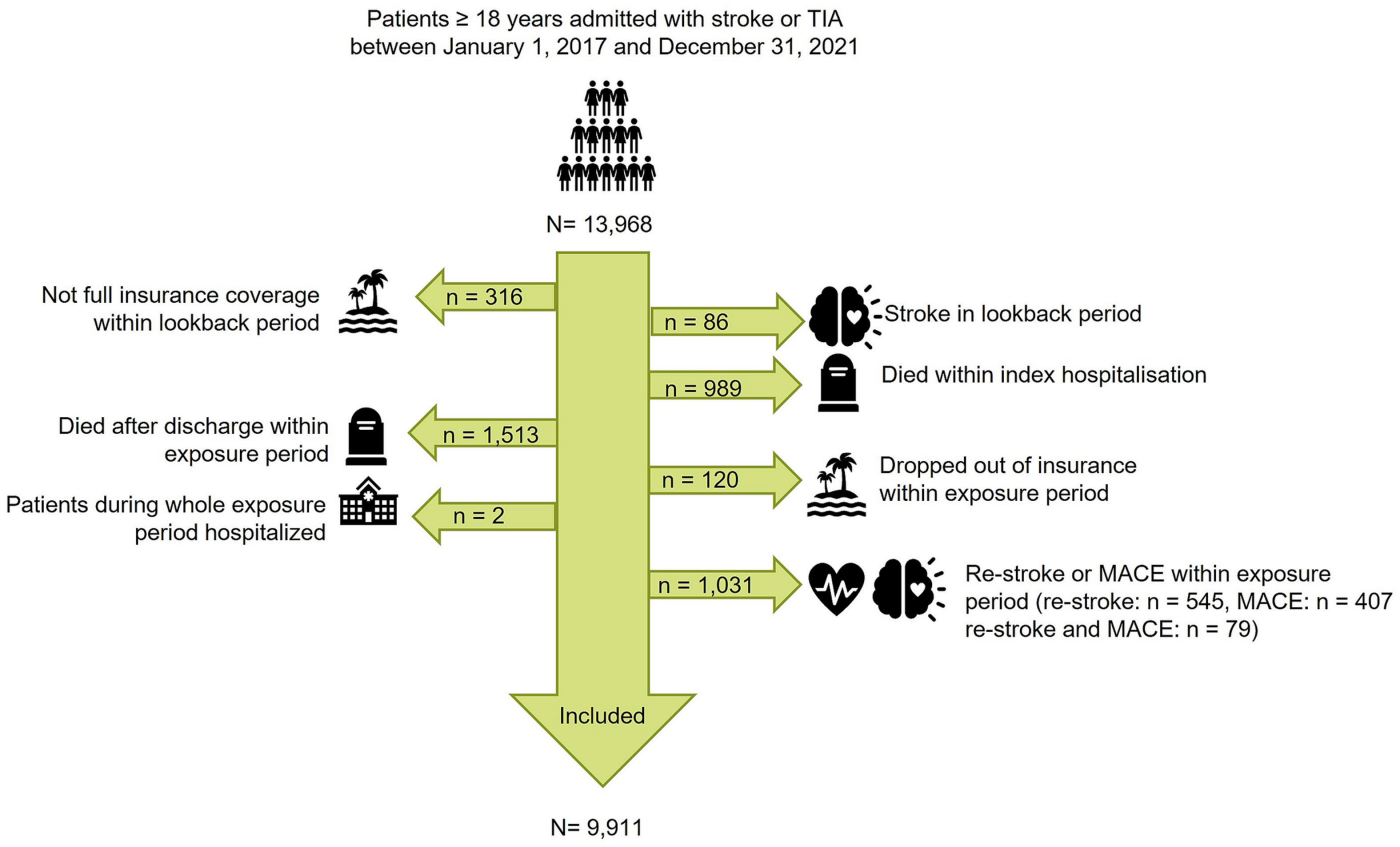
Flow chart of in- and exclusion. For the outcome “Nursing home”: patients in nursing homes before or within the exposure period were excluded. MACE: Major adverse cardiovascular event.

The exposure period began on the day of the hospital discharge (index-date) and ended after 365 days (Figure 2). The numerator of MPR was assessed without supply exceeding 365 days (fixed MPR (fMPR)), as previously described in the literature (12, 13, 15) (Supplementary Figure S1). It was analyzed for each patient and for each predefined medication group. If patients had two or more different medications out of one predefined medication group, the DDD of the single medication were calculated and numbers were summed up to calculate the combined MPR. MPR was truncated to 1 at the patient level. An MPR of 0.80 or more was set as the threshold to define high adherence (15). Non-users (MPR = 0.0) were not included in the calculation of the mean MPR. To define high adherence to combination therapy (aspirin, LLD, antihypertensives) the MPR of each medication group had to have a value of 0.80 or higher. For grouped medication (for example “antiplatelets”) one of the potential substances had to have a value of 0.80 or more to define high adherence.

The MPR was accessed for the medication groups recommended in international medical guidelines for secondary prophylaxis after stroke and TIA (platelet aggregation inhibitors, LLD, antihypertensives) as well as for the medication indicated for patients with ischemic stroke of certain etiology or with certain comorbidities (8, 16, 17). Prescriptions were identified using the anatomical therapeutical chemical (ATC) code (14). It refers to insulins (A10 A) and antidiabetics without insulins (A10 B, summarized as “antidiabetics,” clopidogrel (B01AC04)), acetylsalicylic acid (aspirin, B01AC06) and ticagrelor (B01AC24, summarized as “antiplatelets”), direct thrombin inhibitors (B01AE) and anti-Xa inhibitors (B01AF, summarized as “direct oral anticoagulants (DOAC)”), heparins (B01AB), vitamin K antagonists (B01AA), selective calcium channel blockers with mainly vascular effects (CCB, C08C) and agents acting on the renin-angiotensin system, including combinations (ACEi/ARB, C09, summarized as “antihypertensives”), diuretics (C03), beta-adrenoceptor antagonist (BB, C07), and agents influencing the lipid metabolism (LLD, C09).

### Outcome definition

To prevent possible immortal time bias, exposure and outcome periods were separated (Figure 2). Outcome parameters were assessed from day one after the exposure period (landmark date) until either to the date of death, insurance drop out or end of follow-up whichever came first. The follow-up period ended on August 04, 2024. Outcomes of interest were restroke (I63.-, I64.- and G45.-), all-cause death, major adverse cardiovascular events [(MACE), myocardial infarction (I21.-, I22.-), instable angina (I20.0), peripheral arterial disease (I70.2)] and admission to nursing home. Patients who were already admitted to a nursing home in the lookback or exposure period, were excluded from the outcome analysis for “admission to nursing home”.

### Other variables/covariates

Baseline characteristics of patients were determined at the index date (dismission of hospital) and included sex, age, geographical region and model of health insurance care (standard versus managed care). The medication taken in the last year before the index date was also evaluated. Comorbidities were assessed and the Charlson Comorbidity Index was calculated as a summarized comorbidity index according to Glasheen et al. (18), and measured within the look-back period, including diagnoses during the index hospitalization. The severity of comorbidities was categorized as mild (CCI 1–2), moderate (CCI 3–4), and severe (CCI > 4).

### Statistical analysis

For descriptive statistics we used group comparison tests (Pearson’s chi-squared test for categorical variables and nonparametric analysis of variance for continuous variables). The association between patient characteristics and MPR ≥ 0.80 was estimated by using multivariate logistic regression analysis, adjusted for potential confounders. The association between MPR ≥ 0.80 and clinical outcomes was estimated by using Cox proportional hazards regression models and censored for loss to follow-up. Since evidence supporting thresholds of MPR is limited (19), we additionally modelled MPR in high and low MPR (> 0 to 0.79). Statistical significance was set at the 0.05 level. All analyses were performed using the statistical program R, version 4.3.1 (R Foundation for Statistical Computing, Vienna, Austria).

### Ethics approval

The analysis of data from this study was entirely based on anonymized and aggregated claims data from the Helsana Group and did not fall under the Swiss Federal Act on Research involving Human Beings. Consequently, an approval from the competent ethics committee was not necessary.

## Results

We identified a total of 13,986 patients with stroke or TIA. After exclusion of patients who did not have full insurance coverage within the lookback period (316), had a stroke during the look back period (86), died during the index hospitalization (989), died after discharge within the exposure period (1,513), dropped out of the insurance in the exposure period (120), were hospitalized during the whole exposure period (2) or had a restroke or MACE within the exposure period (1,031), 9,911 patients were included in the present analysis (Figure 2). In-hospital mortality rate of the patient cohort with stroke or TIA was 7.3%. Mortality at the end of the exposure period, one year after index hospitalization, was 18.5%. The median survival of all included patients with stroke or TIA was 2,179 days (95% CI 2,156, 2,216), (6 years) (Supplementary Figure S2). The median follow-up for all patients was 1,044 days (34.3 months), and 629 days (20.7 months) for deceased patients.

### Patient characteristics

Gender was almost evenly distributed in the present cohort: 51.0% of patients were female. The mean age of women and men at index date differed significantly with 76.5 (SD 12.7) versus 71.8 (SD 12.7) years (*p* < 0.001). 56.0% of patients were enrolled in a managed care health insurance model. Of the medication analyzed, the most frequently taken medication within one year before stroke or TIA were ACEi/ARB (49.6%), LLD (34.1%) and BB (33.1%). As a summary measure the Charlson Comorbidity Index showed mild to moderate disease severity with a mean of 2.4 conditions. The most frequent comorbidities were hypertension (68.3%), atrial fibrillation 20.8% and diabetes (18.8%). Table 1 summarizes the baseline characteristics of the study cohort.

**Table 1:** Baseline characteristics of the study patients at index date.

Characteristic	Total	Male	Female	*p*-value
Total, *n* (%)	9,911	4,860 (49.0%)	5,051 (51.0%)	>0.1
Age, mean (SD)	74.2 (12.9)	71.8 (12.7)	76.5 (12.7)	*** ^a^
Age, classes				*** ^c^
< 60	1,355 (13.7%)	850 (17.5%)	505 (10.0%)	
60, < 70	1,652 (16.7%)	983 (20.2%)	669 (13.2%)	
70, < 80	2,973 (30.0%)	1,557 (32.0%)	1,416 (28.0%)	
80, < 90	3,171 (32.0%)	1,243 (25.6%)	1,928 (38.2%)	
≥ 90	760 (7.7%)	227 (4.7%)	533 (10.6%)	
Managed care model				*** ^c^
Standard	4,360 (44.0%)	2,046 (42.1%)	2,314 (45.8%)	
Managed care	5,551 (56.0%)	2,814 (57.9%)	2,737 (54.2%)	
Deductible				*** ^c^
Low	8,544 (86.2%)	3,959 (81.5%)	4,585 (90.8%)	
High	1,367 (13.8%)	901 (18.5%)	466 (9.2%)	
Language region				^c^
German	7,527 (75.9%)	3,720 (76.5%)	3,807 (75.4%)	
French	1,606 (16.2%)	785 (16.2%)	821 (16.3%)	
Italian	778 (7.8%)	355 (7.3%)	423 (8.4%)	
Drugs used within 1 year before index				
Insulins	542 (5.5%)	326 (6.7%)	216 (4.3%)	*** ^b^
Antidiabetics without insulins	1,393 (14.1%)	809 (16.6%)	584 (11.6%)	*** ^b^
Vitamin K antagonists	556 (5.6%)	315 (6.5%)	241 (4.8%)	*** ^b^
Heparine group	541 (5.5%)	277 (5.7%)	264 (5.2%)	^b^
Clopidogrel	568 (5.7%)	315 (6.5%)	253 (5.0%)	** ^b^
Acetylsalicylic acid	3,144 (31.7%)	1,613 (33.2%)	1,531 (30.3%)	** ^b^
Ticagrelor	52 (0.5%)	31 (0.6%)	21 (0.4%)	^b^
Direct thrombin inhibitors	27 (0.3%)	12 (0.2%)	15 (0.3%)	^b^
Anti-Xa inhibitors	1,232 (12.4%)	586 (12.1%)	646 (12.8%)	^b^
Diuretics	1,900 (19.2%)	786 (16.2%)	1,114 (22.1%)	*** ^b^
Beta-adrenoceptor antagonist	3,276 (33.1%)	1,469 (30.2%)	1,807 (35.8%)	*** ^b^
Selective CCB with mainly vascular effects	1,863 (18.8%)	819 (16.9%)	1,044 (20.7%)	*** ^b^
Agents acting on the Renin-angiotensin system, including combinations	4,920 (49.6%)	2,409 (49.6%)	2,511 (49.7%)	^b^
Agents influencing the lipid metabolism	3,375 (34.1%)	1,874 (38.6%)	1,501 (29.7%)	*** ^b^
Comorbidity at index date				
Diabetes	1,865 (18.8%)	1,098 (22.6%)	767 (15.2%)	*** ^b^
Hypertension	6,772 (68.3%)	3,238 (66.6%)	3,534 (70.0%)	*** ^b^
Unstable angina	105 (1.1%)	53 (1.1%)	52 (1.0%)	^b^
Congestive heart failure	840 (8.5%)	417 (8.6%)	423 (8.4%)	^b^
Atrial fibrillation	2,060 (20.8%)	1,012 (20.8%)	1,048 (20.7%)	^b^
Myocardial infarction	229 (2.3%)	151 (3.1%)	78 (1.5%)	*** ^b^
CCI				** ^c^
1–2	6,752 (68.1%)	3,237 (66.6%)	3,515 (69.6%)	
3–4	2,215 (22.3%)	1,140 (23.5%)	1,075 (21.3%)	
> 4	944 (9.5%)	483 (9.9%)	461 (9.1%)	
CCI, mean (SD)	2.4 (1.8)	2.4 (1.8)	2.4 (1.8)	** ^a^

a Kruskal-Wallis test.

b Fisher exact test.

c Chi-Square test.

Significance codes: 0 ‘***’; 0.001 ‘**’; 0.01 ‘*’; 0.05 ‘.’; 0.1 ‘’.

For a sensitivity analysis, we analyzed all rehabilitation and hospital stays in patients with stroke or TIA after the index hospitalization during the one-year exposure period. 1,853 patients (18.7%) had at least one stay in a rehabilitation clinic. The average length of stay in the rehabilitation clinic were 39.0 days (SD 28.0). 18.0% of patients had at least one stay in an acute hospital or psychiatric hospital, the average length of stay was 12.7 days (SD 16.5). The proportion of patients who were treated both in hospital and rehabilitation clinics was 14.2%, with a combined mean length of stay of 60.3 days (SD 40.5) (Supplementary Table S1). For this reason, we excluded all days on which the patient was hospitalized from the denominator of the MPR, as including hospitalization days in the MPR could lead to an underestimation of the MPR, since in Switzerland most hospitals and rehabilitation clinics provide the patient with medication.

### Medication use and medication possession ratio

Among the medication groups examined, LLD had the highest proportion of users (patients with at least one prescription within the exposure year) at 80.4%, followed by ACEi/ARB (62.1%) and aspirin (58.3%) (Table 2). Nearly 28% of patients received at least one prescription for anti-Xa inhibitors during the exposure period and 26.5% one for clopidogrel. Among the aspirin users, the mean MPR was 0.77 (95% CI 0.77, 0.78) and 61.0% of them had an MPR over 0.80, defining high adherence. The mean MPR of LLD- and ACEi/ARB users were 0.88 (95% CI 0.88, 0.89) and 0.79 (95% CI 0.79, 0.80), respectively. High adherence was detected in 78.6% (LLD) and 63.5% (ACEi/ARB) of the users. Among all patients with stroke or TIA, 63.2% had high adherence to LLD and 39.5% had high adherence to ACEi/ARB.

**Table 2:** Medication usage and medication possession ratio (MPR).

Medication	Use *n* (%)	MPR mean (users only) (95% CI)	MPR ≥ 0.80 (users only) %	MPR ≥ 0.80 (all patients) *n* (%)
Insulins	702 (7.1%)	0.607 (0.583, 0.631)	36.2%	254 (2.6%)
Antidiabetics without insulins	1,581 (16.0%)	0.692 (0.676, 0.708)	48.9%	773 (7.8%)
Vitamin K antagonists	533 (5.4%)	0.489 (0.466, 0.511)	16.7%	89 (0.9%)
Heparine group	891 (9.0%)	0.145 (0.130, 0.160)	4.3%	38 (0.4%)
Clopidogrel	2,630 (26.5%)	0.695 (0.683, 0.708)	53.4%	1,404 (14.2%)
Acetylsalicylic acid	5,779 (58.3%)	0.774 (0.767, 0.781)	61.0%	3,523 (35.5%)
Ticagrelor	67 (0.7%)	0.630 (0.549, 0.711)	44.8%	30 (0.3%)
Direct thrombin inhibitors	211 (2.1%)	0.718 (0.680, 0.755)	44.5%	94 (0.9%)
Anti-Xa inhibitors	2,732 (27.6%)	0.682 (0.670, 0.694)	48.4%	1,322 (13.3%)
Diuretics	412 (4.2%)	0.463 (0.434, 0.493)	20.1%	83 (0.8%)
Beta-adrenoceptor antagonist	3,804 (38.4%)	0.415 (0.406, 0.424)	13.8%	526 (5.3%)
Selective CCB with mainly vascular effects	2,890 (29.2%)	0.707 (0.695, 0.719)	54.6%	1,579 (15.9%)
Agents acting on the RAS, including combinations	6,158 (62.1%)	0.794 (0.787, 0.801)	63.5%	3,911 (39.5%)
Agents influencing the lipid metabolism	7,973 (80.4%)	0.881 (0.876, 0.886)	78.6%	6,268 (63.2%)
Combination (ACEi/ARB, LLD, aspirin)	3,298 (33.3%)	0.829 (0.823, 0.835)	38.2%	1,260 (12.7%)

CCB: Calcium channel blockers. RAS: Renin-angiotensin system. ACEi/ARB: Agents acting on the RAS, including combinations. LLD: Agents influencing the lipid metabolism. Aspirin: Acetylsalicylic acid.

When it comes to one of the standard combination therapies for secondary prevention after stroke or TIA (aspirin, ACEi/ARB and LLD), 33.3% of patients had at least received one prescription of all three medications, and 38.2% of those had an MPR of 0.80 or higher.

For a sensitivity analysis, we assessed the number and percentage of patients who had more than one substance out of a predefined medication group. The highest percentages of users with more than one medication out of the medication group were seen in insulins (42.2%), antidiabetics (27.1%), ACEi/ARB (16.1%) and LLD (11.1%) (Supplementary Table S2).

The distributions of medication adherence (ranging from MPR > 0 to 1.0) for the six most often used medication groups in the present patient cohorts (LLD, ACEi/ARB, aspirin, BB, CCB, anti-Xa inhibitors) are depicted in density plots (Supplementary Figure S3).

The association between patient characteristics and adherence to medical secondary prevention was analyzed in a multivariate logistic regression model. Female gender exhibited a significant negative influence on the adherence to LLD (OR 0.63, 95% CI 0.57, 0.68), DOAC (OR 0.84, 95% CI 0.73, 0.96) and Vitamin K antagonists (OR 0.51, 95% CI 0.32, 0.81). History of diabetes, myocardial infarction, unstable angina and hypertension were positively correlated with a high adherence to LLD (Table 3).

**Table 3:** Association between patient characteristics and high medication adherence (MPR ≥ 0.80).

Characteristic	Insulin/antidiabetics (*n* = 947, 9.6%)			Vitamin K antagonists (*n* = 89, 0.9%)			Heparins (*n* = 38, 0.4%)			Antiplatelets (*n* = 4,796, 48.4%)		
	OR	95%-CI	*p*-value	OR	95%-CI	*p*-value	OR	95%-CI	*p*-value	OR	95%-CI	*p*-value
(Intercept)	0.008	(0.005, 0.012)	***	0.016	(0.009, 0.029)	***	0.004	(0.002, 0.010)	***	0.935	(0.816, 1.071)	
Sex (female)	0.893	(0.742, 1.074)		**0.506**	**(0.315, 0.810)**	**	1.115	(0.580, 2.145)		0.930	(0.850, 1.017)	
Age groups												
60–69	0.771	(0.541, 1.097)		**0.418**	**(0.221, 0.791)**	**	0.741	(0.288, 1.911)		**1.243**	**(1.068, 1.446)**	**
70–79	0.757	(0.548, 1.048)	.	**0.254**	**(0.136, 0.473)**	***	0.534	(0.216, 1.317)		**1.286**	**(1.118, 1.479)**	***
80–89	**0.422**	**(0.301, 0.591)**	***	**0.263**	**(0.139, 0.495)**	***	**0.245**	**(0.081, 0.739)**	*	**1.203**	**(1.041, 1.391)**	*
≥ 90	**0.174**	**(0.098, 0.308)**	***	**0.155**	**(0.045, 0.538)**	**	0.174	(0.021, 1.453)		1.023	(0.830, 1.262)	
Managed care	0.979	(0.817, 1.173)		1.453	(0.929, 2.273)		1.354	(0.694, 2.639)		0.986	(0.902, 1.077)	
Comorbidity												
Atrial fibrillation	**0.766**	**(0.609, 0.964)**	*	**2.338**	**(1.418, 3.856)**	***	0.391	(0.116, 1.313)		**0.061**	**(0.052, 0.072)**	***
Congestive heart failure	1.279	(0.937, 1.745)		**1.965**	**(1.046, 3.690)**	*	0.343	(0.077, 1.515)		0.921	(0.759, 1.118)	
Diabetes	**231.962**	**(162.956, 330.191)**	***	1.245	(0.733, 2.114)		0.786	(0.350, 1.767)		**1.354**	**(1.197, 1.532)**	***
Myocardial infarction	0.905	(0.558, 1.469)		**2.909**	**(1.320, 6.410)**	**	2.656	(0.613, 11.508)		0.886	(0.658, 1.193)	
Hypertension	1.015	(0.792, 1.300)		0.868	(0.540, 1.395)		0.651	(0.324, 1.308)		**1.426**	**(1.293, 1.572)**	***
Unstable angina	1.400	(0.690, 2.842)		1.314	(0.300, 5.764)		0.000	(0.000, Inf)		**2.274**	**(1.417, 3.649)**	***
CCI 3–4	0.904	(0.736, 1.110)		1.334	(0.765, 2.325)		**4.828**	**(2.124, 10.973)**	***	1.068	(0.948, 1.202)	
CCI > 4	0.785	(0.589, 1.045)	.	1.465	(0.714, 3.006)		**11.205**	**(4.760, 26.375)**	***	1.004	(0.848, 1.188)	

Characteristic	DOAC (*n* = 1,416, 14.3%)			BB (*n* = 527, 5.3%)			Antihypertensives (*n* = 4,488, 45.3%)			LLD (*n* = 6,269, 63.3%)		
	OR	95%-CI	*p*-value	OR	95%-CI	*p*-value	OR	95%-CI	*p*-value	OR	95%-CI	*p*-value
(Intercept)	0.045	(0.034, 0.059)	***	0.016	(0.011, 0.024)	***	0.153	(0.130, 0.179)	***	1.193	(1.044, 1.364)	**
Sex (female)	**0.837**	**(0.732, 0.958)**	**	**1.324**	**(1.100, 1.595)**	**	0.992	(0.907, 1.085)		**0.625**	**(0.573, 0.682)**	***
Age groups												
60–69	**1.421**	**(1.055, 1.912)**	*	1.000	(0.676, 1.479)		**1.342**	**(1.132, 1.590)**	***	**1.673**	**(1.434, 1.952)**	***
70–79	**1.835**	**(1.401, 2.403)**	***	1.081	(0.759, 1.540)		**1.331**	**(1.140, 1.554)**	***	**1.663**	**(1.446, 1.913)**	***
80–89	1.244	(0.944, 1.641)		0.820	(0.571, 1.178)		**1.250**	**(1.068, 1.464)**	**	**1.291**	**(1.120, 1.489)**	***
≥ 90	**0.605**	**(0.425, 0.860)**	**	**0.536**	**(0.330, 0.871)**	*	1.060	(0.860, 1.306)		**0.568**	**(0.468, 0.690)**	***
Managed care	1.094	(0.958, 1.250)		**0.805**	**(0.672, 0.964)**	*	1.028	(0.942, 1.122)		0.992	(0.910, 1.080)	
Comorbidity												
Atrial fibrillation	**18.421**	**(15.937, 21.293)**	***	**2.862**	**(2.351, 3.483)**	***	0.902	(0.807, 1.008)	.	**0.790**	**(0.708, 0.881)**	***
Congestive heart failure	1.025	(0.828, 1.269)		**1.709**	**(1.312, 2.226)**	***	0.945	(0.800, 1.116)		0.976	(0.826, 1.153)	
Diabetes	0.865	(0.723, 1.034)		**1.579**	**(1.278, 1.951)**	***	**1.350**	**(1.202, 1.516)**	***	**1.364**	**(1.208, 1.540)**	***
Myocardial infarction	0.895	(0.582, 1.376)		0.877	(0.508, 1.513)		0.864	(0.651, 1.147)		**1.632**	**(1.188, 2.243)**	**
Hypertension	1.060	(0.909, 1.236)		**2.467**	**(1.892, 3.215)**	***	**7.160**	**(6.403, 8.005)**	***	**1.718**	**(1.563, 1.888)**	***
Unstable angina	**0.454**	**(0.218, 0.946)**	*	**2.203**	**(1.231, 3.944)**	**	0.798	(0.532, 1.197)		**1.600**	**(1.012, 2.531)**	*
CCI 3–4	0.849	(0.716, 1.007)	.	1.242	(0.994, 1.551)	.	1.044	(0.933, 1.169)		0.998	(0.891, 1.118)	
CCI > 4	**0.779**	**(0.616, 0.986)**	*	1.210	(0.900, 1.626)		0.960	(0.820, 1.124)		**0.754**	**(0.644, 0.881)**	***

CCI: Charlson Comorbidity Score. DOAC: direct oral anticoagulants. BB: Beta-adrenoreceptor antagonists. LLD: agents influencing the lipid metabolism. Total *n* = 9,911 for all models. Significance codes: 0 ‘***’; 0.001 ‘**’; 0.01 ‘*’; 0.05 ‘.’; 0.1 ‘’. Significant results are highlighted in bold.

### Outcomes

Death after the end of the exposure period occurred in 2,580 patients (26.0%). 593 patients (6.0%) had a recurrent event, 412 (5.0%) were admitted to a nursing home for the first time, and 607 (6.1%) had a major cardiovascular event.

Table 4 summarizes the results of the Cox proportional hazards regression analysis assessing the influence of high medication adherence (MPR ≥ 0.80) on clinical outcomes from the beginning of the outcome period until the end of the follow-up. When adjusting for sex, age and comorbidity at index date, high adherence for LLD (HR 0.86, 95% CI 0.79, 0.94) and antidiabetics without insulins (HR 0.81, 95% CI 0.68, 0.96) were statistically significantly associated with a lower likelihood for all-cause death. High adherence to DOAC showed a trend towards a protective effect on survival with an HR of 0.88 (95% CI 0.78, 1.00), as did antihypertensives (HR 0.93, 95% CI 0.85, 1.01). Patients with high MPR for antihypertensives furthermore experienced a trend towards a protective effect against restroke (HR 0.82, 95% CI 0.68, 1.00). On the contrary, high adherence to Heparins [HR 2.27, 95% CI (1.40, 3.66)] was associated with a higher likelihood for MACE.

**Table 4:** Association of high medication adherence with clinical outcomes.

	Death (*n* = 9,911, events = 2,580)			Nursing home (*n* = 8,175, events = 412)			Restroke (*n* = 9,911, events = 593)			MACE (*n* = 9,911, events = 607)		
	HR	95% CI	*p*-value	HR	95% CI	*p*-value	HR	95% CI	*p*-value	HR	95% CI	*p*-value
High medication adherence												
Insulins	1.22	(0.96, 1.54)	.	1.05	(0.56, 1.97)		1.21	(0.74, 1.98)		**1.90**	**(1.36, 2.64)**	***
Antidiabetics without insulins	**0.81**	**(0.68, 0.96)**	*	0.89	(0.58, 1.36)		1.26	(0.89, 1.78)		0.94	(0.71, 1.25)	
Vitamin K antagonists	0.68	(0.43, 1.10)		0.72	(0.18, 2.91)		1.66	(0.82, 3.38)		0.99	(0.44, 2.23)	
Heparine group	**2.27**	**(1.40, 3.66)**	***	2.69	(0.66, 10.94)		0.45	(0.06, 3.22)		0.00	(0.00, Inf)	
Platelet aggregation inhibitors	0.99	(0.90, 1.09)		0.88	(0.69, 1.14)		1.16	(0.94, 1.42)		1.11	(0.91, 1.35)	
DOAC	0.88	(0.78, 1.00)	*	1.09	(0.82, 1.46)		0.98	(0.75, 1.28)		0.99	(0.76, 1.29)	
Antihypertensives	0.93	(0.85, 1.01)	.	0.92	(0.74, 1.15)		0.82	(0.68, 1.00)	*	1.19	(1.00, 1.43)	.
LLD	**0.86**	**(0.79, 0.94)**	***	1.10	(0.88, 1.37)		1.12	(0.93, 1.34)		1.02	(0.85, 1.22)	
Combination (aspirin, LLD, antihypertensives)	0.88	(0.75, 1.04)		**1.52**	**(1.07, 2.15)**	*	1.20	(0.90, 1.59)		0.79	(0.59, 1.04)	.
Age groups												
60–69	**2.97**	**(2.12, 4.16)**	***	**2.71**	**(1.09, 6.72)**	*	1.21	(0.84, 1.74)		**2.49**	**(1.58, 3.93)**	***
70–79	**5.41**	**(3.96, 7.40)**	***	**5.83**	**(2.53, 13.41)**	***	**1.63**	**(1.17, 2.25)**	**	**3.15**	**(2.05, 4.84)**	***
80–89	**12.92**	**(9.49, 17.59)**	***	**19.00**	**(8.37, 43.09)**	***	**1.75**	**(1.26, 2.43)**	***	**3.30**	**(2.14, 5.09)**	***
≥ 90	**28.83**	**(20.98, 39.60)**	***	**34.05**	**(14.59, 79.50)**	***	**2.02**	**(1.35, 3.02)**	***	**2.86**	**(1.72, 4.77)**	***
Sex (female)	**0.83**	**(0.77, 0.90)**	***	**1.52**	**(1.24, 1.87)**	***	0.98	(0.83, 1.16)		**0.68**	**(0.57, 0.80)**	***
Comorbidity												
Atrial fibrillation	**1.29**	**(1.16, 1.43)**	***	1.11	(0.85, 1.45)		**1.30**	**(1.02, 1.65)**	*	0.95	(0.75, 1.21)	
Congestive heart failure	**1.21**	**(1.08, 1.37)**	**	0.94	(0.69, 1.30)		**0.70**	**(0.51, 0.98)**	*	0.92	(0.70, 1.19)	
Diabetes	**1.17**	**(1.04, 1.31)**	**	1.24	(0.93, 1.66)		0.95	(0.73, 1.25)		**1.39**	**(1.11, 1.73)**	**
Myocardial infarction	1.17	(0.93, 1.47)		1.57	(0.94, 2.62)	.	1.34	(0.84, 2.13)		**1.55**	**(1.06, 2.28)**	*
Hypertension	**1.15**	**(1.04, 1.27)**	**	0.93	(0.73, 1.19)		**1.33**	**(1.08, 1.64)**	**	**1.55**	**(1.23, 1.94)**	***
Unstable angina	0.90	(0.62, 1.29)		1.29	(0.60, 2.75)		1.41	(0.75, 2.66)		**1.81**	**(1.09, 3.01)**	*
CCI 3–4	**1.77**	**(1.61, 1.94)**	***	**1.90**	**(1.50, 2.40)**	***	1.00	(0.81, 1.23)		**1.51**	**(1.23, 1.84)**	***
CCI > 4	**2.40**	**(2.14, 2.71)**	***	**2.26**	**(1.66, 3.06)**	***	1.30	(0.99, 1.71)	.	**2.90**	**(2.31, 3.63)**	***

Cox proportional hazards regression analysis. MACE: Major adverse cardiovascular event. DOAC: Direct oral anticoagulants. LLD: agents influencing the lipid metabolism. CCI: Charlson Comorbidity Score. Reference: patients with MPR 0.00–0.79. Significant codes: 0 ‘***’; 0.001 ‘**’; 0.01 ‘*’; 0.05 ‘.’; 0.1 ‘’. Significant results are highlighted in bold.

## Discussion

To the best of our knowledge, the present study is the first to access the medical adherence to secondary prevention after ischemic cerebrovascular disease and its clinical outcomes in Switzerland.

The analyses revealed the following key results: firstly, of all patients with stroke or TIA analysed, high adherence to secondary preventive medication was observed in 63.2% (LLD), 55.4% (antihypertensives) and 50.0% (antiplatelets), respectively. Only one of eight patients was highly adherent to one of the standard drug class combination recommended in the guidelines for stroke without underlying atrial fibrillation (aspirin, ACEi/ARB, LLD). Although medical secondary prevention has been shown to play an important role in improving long-term clinical outcomes in patients with stroke and TIA (3), real life medical care appears to be highly deficient. The finding of suboptimal adherence is consistent with older observational studies. Analyses of data from Australian and Singapore registries until 2014 showed that the proportion of highly adherent patients within the first year after the event ranged from one to two thirds (20, 21). Other studies came to similar results, however, many of those only analyse short time exposure intervals for adherence (22–24). Yet, there is evidence that adherence to secondary prevention decreases with increasing distance from the cardiovascular event (25). Discrepancies in results may, among other things, stem from the heterogeneity in how medication use is operationalized across studies. For example, some studies consider the presence of a prescription as an indicator of medication use without applying established adherence metrics like the MPR or the proportion of days covered (PDC) (23, 26). A very recent claims-based study by Fleet et al. (27) follows a similar descriptive approach, reporting prescribing patterns without quantifying adherence behavior or assessing clinical outcomes. In contrast, our study applies a standardized adherence measure and relates it to multiple clinical endpoints, thereby providing a broader assessment of secondary stroke prevention. Secondly, female patients were 37% less likely to adhere to recommended LLD therapy than men, highlighting a significant gender gap supporting results of recent studies from the US (28) and Italy (29). Future research is needed to understand underlying causes, whether patient-, provider-, or drug-related, to enable interventions that address these differences. Furthermore, a predictive factor for high adherence to antihypertensives, LLD and antiplatelets was increased age (60 to < 90 years). Nonagenarians, however, showed lower adherence to lipid lowering therapy, potentially reflecting altered risk–benefit considerations and a greater sensitivity to adverse drug effects in this age group (30, 31). These are interesting results since a meta-analysis from 2016 (10) analyzing predictive factors for non-adherence to secondary preventative medication after stroke found that all analysed parameters (absent history of atrial fibrillation, disability, polypharmacy and age) were not significantly associated with adherence. Discrepancies in studies might be due to different study populations with unknown or unadjusted confounders, e.g., patient-related factors (for example, concerns about treatment), socioeconomic status (low education level, no settled work status) and disease related factors like reduced cognitive function or poor quality of life as well as institutional aspects like lack of support with medication (10).

Thirdly, high MPR for LLD and antidiabetics were significantly associated with a reduced risk for all-cause death, showing a risk reduction of 14 and 19%, respectively. This is in line with previous studies, although the effect is not as pronounced in our analysis (20, 23). Furthermore, high adherence to DOAC and antihypertensives showed a tendency towards a protective effect on all-cause death. However, in our patient cohort the confidence interval’s boundaries were around 1, indicating a lack of strong evidence against the null hypothesis, even after adjusting for important confounders like atrial fibrillation, which was previously shown to lead to a 54% higher risk for stroke recurrence (32). In a meta-analysis on real-world adherence to DOAC in patients with atrial fibrillation, the pooled HR of three studies showed a 39% increased risk of stroke in non-adherent patients (33). Antihypertensives were the only medication group for which a high MPR showed a trend towards a protective effect against restroke. Interestingly, and partly in contradiction to other study results (3, 20, 33–35), high adherence to secondary medical prevention had no significant protective association in other medication/ outcome combinations, i.e., restroke, admission to nursing home and MACE in our analysis.

Discrepancies in outcome results might be due to immortal time bias, that can occur when the MPR is calculated without dividing exposure and outcome periods (34). Thus, it may have led to an overestimation of the protective effects of high MPR on secondary vascular events or all-cause death in previous studies. Additionally, the risk of restroke is highest within the first year after stroke (1, 32, 36), so the protective effect of DOAC for example, might be underestimated when excluding patients with restroke in the exposure period as we did in our analysis. Further causes could be differences in study cohorts and unadjusted confounders such as stroke severity and disability.

### Clinical and health economic implications

Our results demonstrate the importance to support stewardship measures to improve adherence to secondary prevention after stroke or TIA. A lot of those measures are widely accepted and with proven potential to optimize adherence to medication (5).

Dalli et al. (37) provided evidence that patients’ understanding of stroke prevention medications and medication adherence were positively correlated. Cochrane meta-analysis (38) found that intensification of patient care interventions improves short- and long-term medication adherence to LLD. And Tsai et al., have shown that a prescription at discharge improves long-term adherence for secondary stroke prevention (39).

Moreover, non-adherence to secondary prevention after ischemic cerebrovascular disease is not only problematic from a health care quality point of view but may also be problematic in terms of healthcare expenditures (40, 41). For example, in a claims-based study by Wyl et al. (40), lower health care expenditures were observed when patients received guideline recommended 4-class secondary prophylaxis after myocardial infarction. Further studies are needed to analyse cost-efficacy in secondary stroke prevention.

### Strengths and limitations

The present study has several important strengths. It is based on a large study population that includes about 1.4 million mandatory health insurance customers from all parts of Switzerland. The study therefore most likely reflects the reality of everyday clinical practice well and provides valuable real-world evidence—in contrast to randomized clinical trials (RCT), which often do not adequately consider patients with high comorbidity scores or at high age (42). Previous population-based studies using Swiss Helsana claims data with national extrapolation (43, 44) have shown a close agreement between derived estimates and official statistics from the Swiss Federal Statistical Office. This empirical concordance supports the representativeness of the Helsana-insured population for epidemiological analyses, although insurer-specific differences cannot be fully excluded. Additionally, patients who live in nursing homes are often excluded in analyses (21), although they comprise an important and relatively large group within the cohort of stroke patients and were therefore included in the present analysis. In addition, medication adherence is not the subject of the investigation in most RCT and is also usually not a problem due to controlled settings by study investigators.

However, there are also certain limitations in our study related to the observational study design and the nature of claims data: First, we had no information about other secondary prevention measures like life-style changes (sports, nutrition, smoking etc.), that might also have an influence on clinical outcomes after ICD (45). Second, several patient related potential confounders were not assessable in our data and therefore we could not adjust for them, for example, for stroke severity (46) or—subtypes, socioeconomic status, contraindications to medication (e.g., risk of bleeding) or adverse drug reactions. Further research on adherence is needed and would be most valuable if data from healthcare insurances and from clinical settings could be linked anonymously.

Regarding MPR calculation, our study has both important strengths and few limitations that cannot be overcome by claims data. As Tang et al. aptly stated in 2017, there is no standard operational definition for adherence, and methods used to calculate it vary significantly between studies, even when they use the same terminology (15). In our analysis, we therefore endeavoured to clearly describe all operational definitions and apply recommended measures (47), including, among other things, dealing with double medication within a drug class and exclusion of hospital days from the MPR denominator.

Moreover, as with all claims-based analyses, dispensed prescriptions indicate availability rather than actual ingestion. Consequently, true patient-level adherence may be overestimated, which could attenuate the observed associations between adherence and clinical outcomes. Also, the clinical effects of medical therapy are not accessible with claims data (for example, we do not know the blood lipid level of patients, either in single or in combination therapy). In addition, different substances which we analysed in one drug class may influence outcomes: Within the group of LLD, relatively novel PSCK9 inhibitors could reduce the risk for early recurrent stroke in patients with intracranial atherosclerosis in comparison to statins and/ or ezetimibe (48).

## Conclusion

A remarkable proportion of patients did not receive medical secondary prophylaxis after ischemic cerebrovascular disease and those who received it had often only moderate medication adherence. Inadequate secondary prophylaxis to LLD was associated with an increased all-cause death rate and therefore efforts are needed to raise awareness among healthcare professionals and patients about the importance of secondary prevention in patients with ischemic cerebrovascular disease.

## Data Availability

The datasets analysed during the current study are not publicly available due to reasons of individual privacy, legal and regulatory affairs, but are available from the corresponding author on reasonable request. Requests to access the datasets should be directed to sabrina.stollberg@helsana.ch.
