# Supplementary material for: Medication adherence to secondary prevention after ischemic cerebrovascular disease: a real-world outcomes analysis
Source: Front Neurol. 2026 Mar 6;17:1764948. doi: 10.3389/fneur.2026.1764948 (PMC13002439; doi:10.3389/fneur.2026.1764948)
Supplement: Supplementary file 1 [file Data_Sheet_1.DOCX]

**Supplemental Material**

Intended for publication as an online data supplement.

*Supplementary Figures*

Figure S1: Fixed MPR (fMPR) without supply exceeding 365 days.


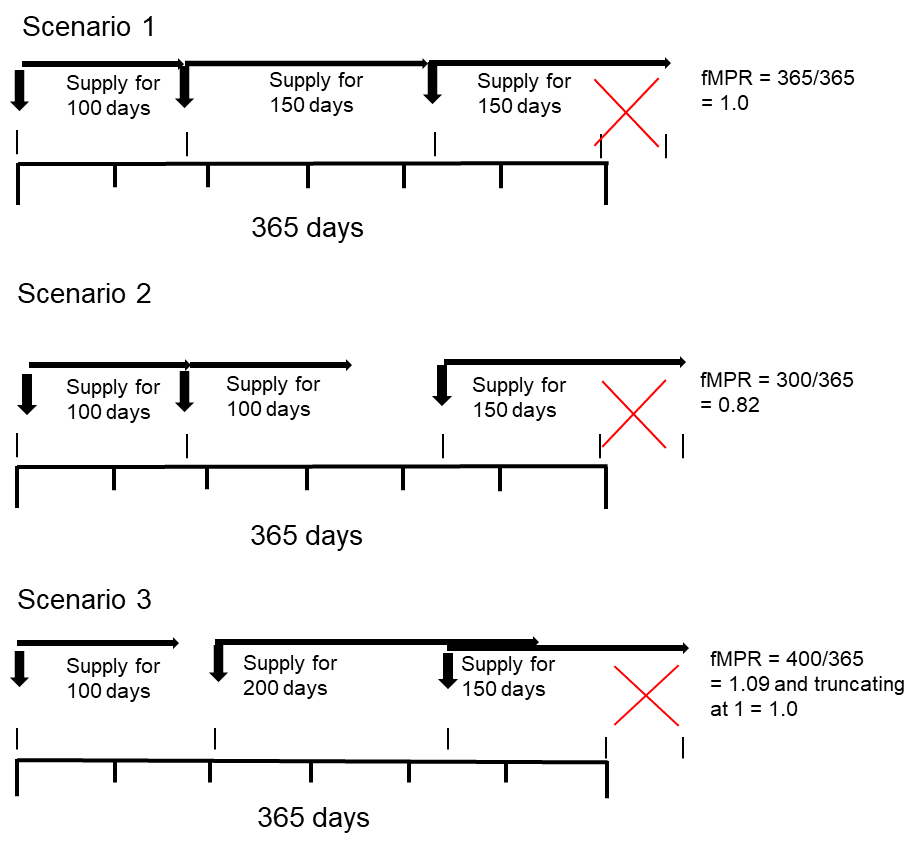
fMPR: fixed MPR. Three of the possible scenarios presented as examples. Supply in DDD.

Figure S2: Kaplan-Meier curve for all patients with stroke or TIA


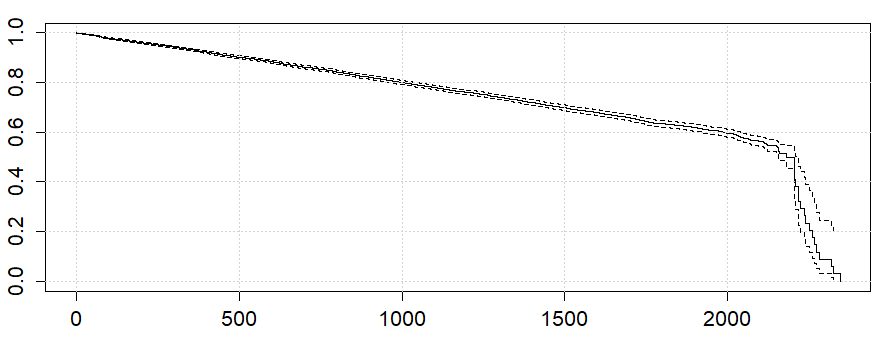


Probability of survival

Time (days)

Survival probability --- 95% Confidence interval

Figure S3: Density plots: Medication possession rate (MPR)


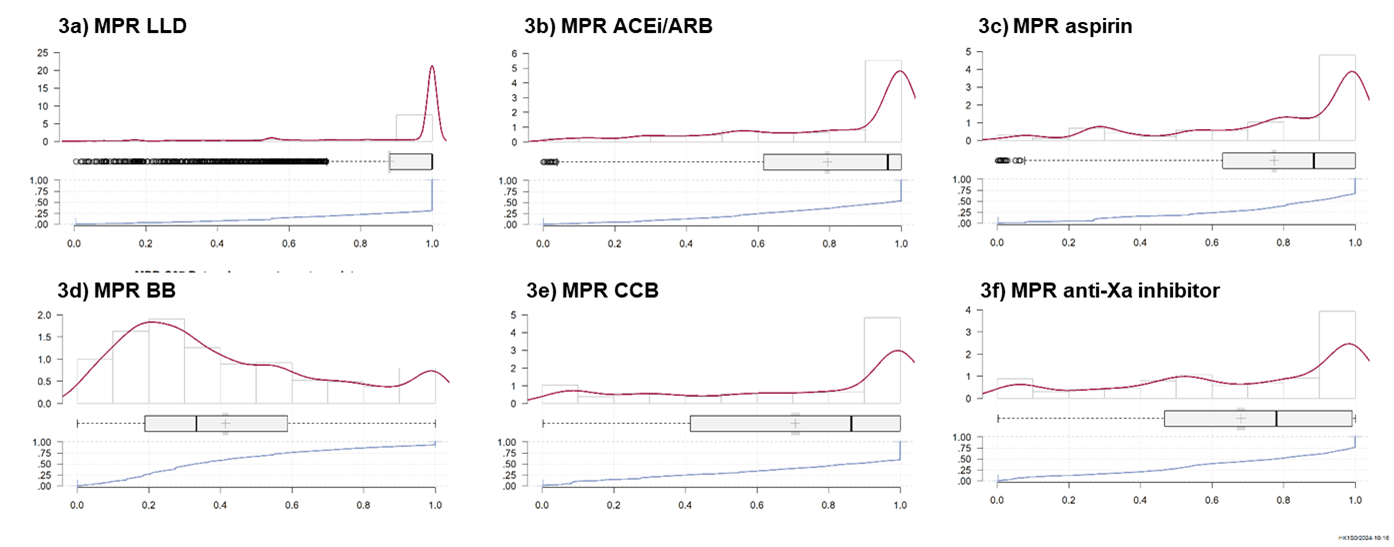


LLD: agents influencing the lipid metabolism. ACEi/ARB: agents acting on the renin-angiotensin system, including combinations. Aspirin: acetylsalicylic acid. BB: beta-adrenoreceptor antagonists. CCB: selective calcium channel blockers with mainly vascular effects.

---- Density Function of MPR

---- Empirical Cumulative Function

+ Mean

Median

*Supplementary Tables*

Table S1: Rehabilitation and Hospital stays during observation period

|  | **Rehabilitation stays** | **Hospital stays** | **Rehabilitation and**  **hospital stays** |
| --- | --- | --- | --- |
| **Total** (n / %) | 1,853 / 18.7% | 1,788 / 18.0% | 1,412 / 14.2% |
| **Female** (n / %) | 932 / 50.3% | 925 / 51.7% | 725 / 51.3% |
| **CCI** (mean, SD) | 2.7 (1.8) | 2.3 (1.7) | 3.1 (2.2) |
| **Age** (mean, SD) | 74.4 (12.8) | 75.4 (12.8) | 75.6 (12.2) |
| **Length of stay** (mean, SD) | 39.0 (28.0) | 12.7 (16.5) | 60.3 (40.5) |

CCI: Charlson Comorbidity Index

Table S2: Number of different substances prescribed within one medication group (within exposure period)

|  | 1 substance (= user) | 2 substances | 3 substances | ≥ 3 substances | Percent of users with ≥ 2 substances |
| --- | --- | --- | --- | --- | --- |
| Insulins | 353 (3.6%) | 297 (3.0%) | 49 (0.5%) | 4 (0.0%) | 42.2% |
| Antidiabetics without insulins | 942 (9.5%) | 429 (4.3%) | 158 (1.6%) | 52 (0.5%) | 27.1% |
| Vitamin K antagonists | 529 (5.3%) | 4 (0.0%) | 0 (0.0%) | 0 (0.0%) | 0.8% |
| Heparine group | 847 (8.5%) | 43 (0.4%) | 3 (0.0%) | 0 (0.0%) | 4.8% |
| Clopidogrel | 2,630 (26.5%) | 0 (0.0%) | 0 (0.0%) | 0 (0.0%) | NA |
| Acetylsalicylic acid | 5,779 (58.3%) | 0 (0.0%) | 0 (0.0%) | 0 (0.0%) | NA |
| Ticagrelor | 67 (0.7%) | 0 (0.0%) | 0 (0.0%) | 0 (0.0%) | NA |
| Direct thrombin inhibitors | 211 (2.1%) | 0 (0.0%) | 0 (0.0%) | 0 (0.0%) | 0.0% |
| Anti-Xa inhibitors | 2,630 (26.5%) | 101 (1.0%) | 1 (0.0%) | 0 (0.0%) | 3.7% |
| Diuretics | 404 (4.1%) | 8 (0.1%) | 0 (0.0%) | 0 (0.0%) | 1.9% |
| Beta-adrenoceptor antagonist | 3,657 (36.9%) | 139 (1.4%) | 9 (0.1%) | 0 (0.0%) | 3.7% |
| Selective CCB with mainly vascular effects | 2,687 (27.1%) | 194 (2.0%) | 8 (0.1%) | 1 (0.0%) | 6.7% |
| Agents acting on the RAS, including combinations | 5,011 (50.6%) | 989 (10.0%) | 132 (1.3%) | 26 (0.3%) | 16.1% |
| Agents influencing the lipid metabolism | 6,864 (69.3%) | 885 (8.9%) | 194 (2.0%) | 30 (0.3%) | 11.1% |

NA: Not applicable. CCB: Calcium channel blockers. RAS: Renin-angiotensin system.
